# Issues and opportunities of digital phenotyping: ecological momentary assessment and behavioral sensing in protecting the young from suicide

**DOI:** 10.3389/fpsyg.2023.1103703

**Published:** 2023-06-27

**Authors:** Silvia Francesca Maria Pizzoli, Dario Monzani, Lorenzo Conti, Giulia Ferraris, Roberto Grasso, Gabriella Pravettoni

**Affiliations:** ^1^Department of Oncology and Hemato-Oncology, University of Milan, Milan, Italy; ^2^Department of Psychology, Catholic University of the Sacred Heart,, Milan, Italy; ^3^Department of Psychology, Educational Science and Human Movement, University of Palermo, Palermo, Italy; ^4^Applied Research Division for Cognitive and Psychological Science, IEO European Institute of Oncology IRCCS, Milan, Italy

**Keywords:** digital phenotyping, ecological momentary assessment, new technologies, suicide risk, adolescence, youth, mHeath applications

## Abstract

Digital phenotyping refers to the collection of real-time biometric and personal data on digital tools, mainly smartphones, and wearables, to measure behaviors and variables that can be used as a proxy for complex psychophysiological conditions. Digital phenotyping might be used for diagnosis, clinical assessment, predicting changes and trajectories in psychological clinical conditions, and delivering tailored interventions according to individual real-time data. Recent works pointed out the possibility of using such an approach in the field of suicide risk in high-suicide-risk patients. Among the possible targets of such interventions, adolescence might be a population of interest, since they display higher odds of committing suicide and impulsive behaviors. The present work systematizes the available evidence of the data that might be used for digital phenotyping in the field of adolescent suicide and provides insight into possible personalized approaches for monitoring and treating suicidal risk or predicting risk trajectories. Specifically, the authors first define the field of digital phenotyping and its features, secondly, they organize the available literature to gather all the digital indexes (active and passive data) that can provide reliable information on the increase in the suicidal odds, lastly, they discuss the challenges and future directions of such an approach, together with its ethical implications.

## Introduction

The fast development of new technologies and the exponentially growing adoption of personal digital devices have been providing new opportunities for research and clinical psychology practice. Specifically, traditional psychological assessment methodologies, such as self-report measures or ability-based tests, can now be integrated with the automatic, unobtrusive, and timely collection of personal information. Digital phenotyping is an excellent example of the possible combination of traditional and digital data to acquire real-time information about one individual’s psychological functioning, mental well-being, and physical health. Onboard sensors and phone logs embedded in smartphones allow the collection of continuous and fine-grained information about people’s daily activities, social interactions, and mobility patterns (Harari et al., 2016, 2017).

Defined as “*moment-by-moment quantification of the individual-level human phenotype in situ using data from personal digital devices*” (Torous et al., 2016) digital phenotyping is indeed an attempt to draw out individual phenotypes from the vast amounts of data that an individual generates via their interactions with digital devices (Birk and Samuel, 2022). The term was first coined in the field of psychological and psychiatric research (Torous et al., 2016) and it was proposed as a means to gather data on social and behavioral manifestations of schizophrenia. However, as will be underlined in the following, digital phenotyping can have many different applications in the psychological field, from the study of personality (Montag and Elhai, 2019) to the identification of unhealthy lifestyles (Skinner et al., 2017).

The aim of this paper is to present a brief overview of digital phenotyping and to outline its possible application to forecast risk for suicidal ideation (SI) or attempts (SA) over time in adolescents. Specifically, suicide risk forecasting might be an illustrative example of how digital phenotyping can be applied in research and clinical practice.

A huge amount of digital data can be used to differentiate or quantify phenotypes, namely cognitive functioning, behavioral and sleep patterns, physical mobility, speech production, social interactions, and many more (Onnela, 2021). This information can be collected with different modalities, ranging from implicit monitoring, thanks to the adoption of different types of sensors, to an explicit collection of data via patient-reported outcomes (Sequeira et al., 2019). The combination of these two sources yields an impressive quantity of valuable information to characterize social behavior and develop the individual’s digital phenotype (Barrigon et al., 2019).

Data in digital phenotyping are usually divided into active (i.e., data generated by active participation, such as filling out a survey or a self-report questionnaire) and passive (i.e., data that are collected without actively involving the participant) data (Onnela, 2021). A more recent definition of digital phenotyping data (Birk and Samuel, 2022) suggests a further distinction of digital data: sensor data generated by sensors built into smartphones (e.g., Bluetooth and GPS to capture temperature, speed, distance, location), activity data generated by the individual’s activity on their digital devices (e.g., messages, screen activity logs, calls) including also self-report and questionnaires (e.g., Ecological momentary assessment and diary studies), and social media data generated by posts on social media (e.g., language used and timing of posts on Facebook, Twitter, Instagram etc.).

The first implementation of digital phenotyping was limited to the use of mobile devices for administrating web-based surveys, writing up electronic diaries, or collecting physiological data that required the direct involvement of individuals (Radhakrishnan et al., 2020). Later on, thanks to the improvements in digital technology, smartphones and wearables have allowed for collecting much larger amounts of data, at relative convenience to the patient. Among the other potential applications, digital phenotyping has already been applied to the evaluation of personality (Marengo and Montag, 2020), psycho-diagnostics of internet use disorders (Montag and Rumpf, 2021), assessment of unhealthy lifestyles (Skinner et al., 2017) and mental health (Brietzke et al., 2019). Put in other words, digital phenotyping allows one to acquire a more detailed pattern of one’s distinctive features by collecting information coming from different data sources. By considering the broad classification of data sources for personality psychology assessment (Cattell, 1958; Block, 1993; Cervone and Pervin, 2018), digital phenotyping can target: (a) L-data, namely information that can be obtained from a person’s life history or life record; (b) O-data, namely observation and information provided by external observers; (c) namely information acquired through experimental procedures aimed at measuring one’s performance on specific tasks; and (d) S-data, namely information that, like self-report measures, are directly reported by the target individual.

The characterization of digital phenotype assumes a clinical relevance in those populations that require constant monitoring of their psychological health.

### Suicidal risk in adolescence: a testing ground for digital phenotyping

A field of interest for the use of digital phenotyping might be youth suicide and the collection of data through the use of digital tools, as well as providing monitoring of the possible fluctuation of clinical symptoms, would be particularly suitable for youth individuals accustomed to the use of technological devices.

Suicide is one of the leading causes of death amongst adolescents and young adults (i.e., 15–29 years old), accounting for 8.5% of all deaths worldwide (World Health Organization, 2014). The developmental shift from childhood to adolescence marks a sharp increase in emotional and behavioural problems that, in combination with individual and environmental risk factors (e.g., mood disorders, preoccupation with death, family history of psychopathology or suicidal behavior (SB), poverty, family discord, and exposure to abuse or neglect) might contribute to causing suicidal thoughts and behaviours (Pfeffer, 1997; Hawton et al., 2003; de Leo and Heller, 2004; Tishler et al., 2007). Noteworthy, during the last two decades, there has been an increase in youth suicide, and adolescents appear to be particularly exposed to the onset of suicidal thoughts and behaviors (Nugent et al., 2022).

Unfortunately, our understanding of suicide among adolescents, including the ability to predict and prevent it, has been hindered by a lack of information about the basic nature of SI (i.e., thoughts) and behaviour (i.e., attempts and actual behaviours) (Brown et al., 2022).

Since digital phenotyping provides real-time characterization and quantification of human behavior *in situ* (Onnela and Rauch, 2016; Bidargaddi et al., 2017; Torous et al., 2017), it might offer a powerful approach to forecasting risk for SI or attempts over time. Given that SI and related outcomes are heterogeneous and time-varying, digital phenotyping methods making use of smartphones and wearables, might accurately detect fluctuations in suicidal thoughts and behaviours as well as guide the delivery of timely support, particularly during high-risk episodes.

### Active and passive data for digital phenotyping in the field of suicidal risk

Unlike the more traditional clinical rating scales, digital phenotyping and ecological momentary assessment (EMA) allows rapid changes in suicide risk to be measured through real-time monitoring of changes in thoughts and behaviors over time. As previously stated, data collected by digital phenotyping can be classified into two main categories: active data which requires the direct involvement of the subject; and passive data, usually collected without any participation or action from the subject (Orsolini et al., 2020). The following section provides an overview of the type of data that might be used or have already been used in the field of digital phenotyping for suicidal risks in adolescents.

#### Active data

Active data refers to the information generated with the involvement of direct and active participation of users. They consist of repeated sampling of an individual’s behavior, experiences, and mood in real-time, in a natural and regular environment.

With the advancement of smartphone technologies, acquiring active data through user input has become more feasible and intrinsically ecological, since its use is widely spread within the population, especially among the youngest, facilitating the uptake of such technologies. Moreover, it has been suggested that active participation in tracking mood could constitute an intervention in itself since it favors empowerment and promotes self-engagement or greater responsibility (Brietzke et al., 2019). Examples of active data include online surveys and questionnaires, audio diaries, or using the phone to carry out cognitive assessments (Onnela, 2021).

##### Survey and EMA

Surveys represent the most adopted instrument to collect active data and a direct way to investigate the individual’s behavior, and rely on the use of validated questionnaires already used in clinical settings. Questionnaires on depression, hopelessness, substance use or stressful and traumatic life event, difficult familiar experiences, might inform on a broad range of risk factors for suicide (Brent et al., 1993; Khasakhala et al., 2013; Cha et al., 2019; Brivio et al., 2021). However, a possible limitation concerns the impossibility of extracting information that is beyond people’s awareness.

Another method of capturing patient-reported data is via EMA, which involves repeated sampling of behavioral aspects, experiences, and moods in real time in one’s regular environment. EMA data can include a range of variables, including questions on a patient’s mood, physical health, or behavioral information (Sequeira et al., 2019). In particular, studies that collected EMA provided important evidence related to the presence of fluctuations in SI, highlighting that suicidal thoughts are dynamic over short periods (Hallensleben et al., 2017; Czyz et al., 2019). Specific EMA items related to the suicide risk can investigate the suicide desire and suicide intent, the inability to keep self-safe, the sense of hopelessness, and emotional dysregulation (Czyz et al., 2022). A growing number of EMA and daily diary studies have revealed important insights about self-injurious thoughts and behaviors occurring in daily life, including nonsuicidal self-injury (NSSI) and its underlying functions (Czyz et al., 2022). Czyz et al. (2018) adopted a daily diary design to examine near-term risk factors for suicide ideation in adolescents over 28 days post-hospitalization, founding that the adoption of these tools was feasible and acceptable among adolescents during this high-risk period (Sequeira et al., 2019).

##### Cognitive and behavioural task

Another way to collect relevant active data consists of the administration of behavioural tasks, that can be digitally administered inside of digital games or challenging activities. Indeed, behavioral tasks are a means to assess suicide risk beyond self-report and can measure objectively features such as impulsiveness, that has already been associated with suicide risk (Cha et al., 2019; Ballard et al., 2021). Indeed, classical cognitive tests can detect specific cognitive features of suicidal attempters. The performances at the Iowa Gambling Task, the Stroop test, the Go/No-Go task, and the Wisconsin Card Sorting Task highlighted that suicide attempters showed less cognitive flexibility, more impulsivity, and worse memory and attention than non-attempters (Keilp et al., 2014; Cha et al., 2019; Zelazny et al., 2019). Other suicide-specific behavioural measures can be applied to identify potential implicit biases for suicide-related content without relying on self-report measures. An interesting example of suicide-specific measure is the Implicit Association Task, that has already been applied to measure associations between suicidal and self-injury behaviours and death and life categories (Nock et al., 2009; Randall et al., 2013) or SI (Price et al., 2014). Furthermore, another way to collect active data of behavioural tasks is using gamified mobile cognitive tests. Introducing a standard experimental task into a pleasant-to-use game, helps to improve both participant engagement and retention (Liu et al., 2021). Overall, such risk factors can be digitally measured in a cost-saving way, providing reliable measures of features that are unlikely to rapidly change over time.

#### Passive data

Passive data refers to information that can be extracted from different devices without the active involvement of the user. They allow for removing the response burden typically involved in active self-monitoring (Skinner et al., 2017), storing up several objective measurements of social, behavioral, and cognitive functioning in real-life settings. Moreover, passive data can also facilitate the identification of clinical symptoms that may be outside the patient’s awareness (e.g., suicide risk). One of the most used and diffused strategies for the acquisition of information is mobile passive monitoring (MPM), whereby data is collected through the smartphone (Jagesar et al., 2021). Most current smartphones incorporate sensors for passive data acquisition related to the physiological status, social behaviour, and contextual information. Other examples are provided by different wearables, such as smart-watches or actigraphs, that have sensors and functionality similar to smartphones, and can also be used as passive data collection instruments.

##### Smartphone sensor and application

Information can be extracted from smartphones and transformed into biopsychosocial measures. These data usually include the detection of an individual’s location through the global positioning systems, analysis of movement thanks to the accelerometers, orientation and angular velocity through gyroscopes and level of illumination from ambient light sensors. A recent study (Huckins et al., 2020) included smartphone data on the locations visited, sedentary time and duration of phone usage to assess mental health in college students during the pandemic. Smartphones also collect contextual information through the analysis of environmental conditions, such as external weather, and communication logs, allowing to examine voice calls, text messages and even spontaneous speech. Also, the keyboard interaction can provide relevant information about reaction time and attention (Sequeira et al., 2019). Smartphones can also passively monitor self-management behavior patterns related to physical activity, sleep cycles, drugs adherence, eating behaviours, heart rate variations, hand movements, medication absorption, or body temperature (Huckins et al., 2020; Radhakrishnan et al., 2020). Moreover, digital phenotyping, using smartphones, seems to have a useful application also in the field of psychiatry and clinical psychology. Passive smartphone-based sensor data were extracted and used, for example, to identify social anxiety symptoms, to predict relapses in schizophrenia and in depressive disorders (Orsolini et al., 2020).

##### Wearables and electronical devices

Different types of wearables can be adopted to collect passive data, their main feature is the possibility of being worn by the subject without any discomfort, allowing the collection of specific information (Vijayan et al., 2021). Actigraphs, for example, are effective tools, usually placed on the wrist, that can be used to reliably estimate an individual’s physical activity and to objectively assess sleep–wake activity (Smith et al., 2018; Ballard et al., 2021). In particular, the analysis of the sleep cycles in high-risk suicidal populations seems crucial since poor quality of sleep was found to be related to SI (Littlewood et al., 2019). The diffusion of innovative technological devices can, also, allow the extraction of new behavioural passive data. An example is given by eye-tracking that, through the use of a webcam or glasses, can provide data related to the shifting of attention and user’s activation on the smartphones’ screens. Another powerful tool is represented by digital recorders, such as electronically activated recorders (EAR) (Kaplan et al., 2020) that can register and store acoustic information from the social context of the users and inform on adolescent social environment and behaviour (Nugent et al., 2022). This latter case can indeed inform on the level of social isolation or interpersonal conflicts, which are important contextual factors for suicide risk (Nock et al., 2009).

##### Social media

Given the huge diffusion of social media platforms among the entire population, several digital footprints are daily produced. Status updates, the number of pictures posted, and also text-mining analysis of posted comments are used to gain insights into individuals’ traits, preferences and likely future behaviour (Montag and Rumpf, 2021). The processing of natural language with software able to analyze word use can also inform on the presence of signs of depression and SI, even when they are not explicitly reported in the texts (Fernández-Cabana et al., 2013; Lumontod and Lumontod, 2020). For example, Linguistic Inquiry and Word Count (LIWC) is a word counting software allowing automated and computerised natural language processing of text. Specifically, it can provide information regarding cognitive, emotional and social content in people’s speech samples. Through LIWc, it is possible to identify distinctive features of suicidal thoughts (Stirman and Pennebaker, 2001; Fernández-Cabana et al., 2013). Plus, machine learning and artificial intelligence had already been applied in trying to assess suicidal risk from social network (Castillo-Sánchez et al., 2020; de Oliveira et al., 2022). Psycholinguistic analysis of posts and tweets had also already been applied to assess emotion expression and somatosensory processes in the context of traumatic experiences (Monzani et al., 2021).

## Challenges and future directions

The case of digital phenotyping in the field of suicidal risk in adolescence can be a good example of the importance of combining active and passive data in studying and predicting the risk trajectory of complex human behaviors. Plus, digital phenotyping might integrate the detection of fast-changing features (such as the actual state of isolation or feelings) with the measurements of more stable traits (such as personality, mood states, and impulsivity). The integration of different sources of data might indeed provide a tailored prediction of suicidal risk, taking into account different features of the individual. Furthermore, the monitoring through passive data would be non-intrusive, ecological, and cost-saving.

Another interesting point of strength and future application for digital phenotyping relates to the concept of connectedness as a preventive and protective factor for suicide (Zareian and Klonsky, 2020).

Connectedness refers to the state of being connected and having relationships with relevant individuals or communities and it has been shown to reduce SA and SI (Resnick et al., 1997), as well as decrease depressive symptoms (Czyz et al., 2012) in adolescents. The connectedness construct might then be coupled with the potentialities of mHealth applications and technologies in the field of suicide prevention and treatment (Costanza et al., 2018). mHealth approaches indeed give the possibility of reaching people in a fast, cost-saving, and ecological manner through web applications and smartphone applications. The connectedness might then be extended to peers, families, communities, and social contact in general, as well as to therapeutic support and programs. Future studies might combine risk profiling with digital phenotyping and a preventive approach with the improvement of connectedness, via dedicated chats or digital platforms, with relevant individuals (peers and family) and healthcare professionals. The timing for specific alerts to improve connections might also be tailored according to data that emerged from the digital phenotyping. In the field of emergency departments for suicide, the use of mHealth has indeed already been proposed as a tool for establishing continuous contact between patients and caregivers and delivering personalized information on treatments and symptoms (Khan and Costanza, 2018).

Despite these advantages, the psychological and ethical implications of applying digital phenotyping in psychological research and clinical practice have been seldom addressed. From a psychological perspective, “observer effect” or “Hawthorne effect” (Landsberger, 1958; Franke and Kaul, 1978) might arise due to digital phenotyping. Specifically, people might modify their behaviors in response to their knowledge of being observed. The observer effect might have relevant and strong consequences on target behaviors considering that observing or recording a behavior with the person’s awareness (i.e., monitoring of behavior by others without feedback) has been considered *per se* a behavioral change technique (Michie et al., 2013). Its effect on conduct might be even more substantial in the case of digital phenotyping because people are aware of being monitored constantly over time.

Digital phenotyping might have relevant ethical implications as well. For example, broader concerns of safety and privacy might arise because digital phenotyping may target diverse sources of data, such as location data and social media, raising possible concerns because of the huge amount of data and related data protection measures (Keller et al., 2020). A recent contribution by Martinez-Martin et al. (2021) developed consensus statements focusing on key areas of ethical issues and regulation of digital phenotyping in the mental health field that should be kept into consideration. They reported strong agreement for five major ethical concerns: (a) transparency (e.g., explanations of the whole process and its associated risks should be provided); (b) consent (e.g., informed consent should be required before collecting personal data through digital phenotyping); (c) data security and privacy (e.g., data and main findings that can allow the identification of individuals should not be collected, processed or shared without the informed consent); (d) fairness (e.g., promote collaborative research to establish effective methods to minimize bias or discrimination when applying digital phenotyping); and (e) accountability (e.g., design and implementation of digital phenotyping methods should be assessed for any possible ethical issue by an independent and interdisciplinary board).

## Data availability statement

The original contributions presented in the study are included in the article/supplementary material, further inquiries can be directed to the corresponding author.

## Author contributions

SP, DM, and LC conceived and discussed the initial idea. SP, DM, LC, and GF wrote the first draft of the manuscript. GF and RG contributed to the manuscript with discussion and insights. GP supervised the entire process and provided feedback on the topic and the structure of the sections of the manuscript. All authors contributed to the article and approved the submitted version.

## Conflict of interest

The authors declare that the research was conducted in the absence of any commercial or financial relationships that could be construed as a potential conflict of interest.

## Publisher’s note

All claims expressed in this article are solely those of the authors and do not necessarily represent those of their affiliated organizations, or those of the publisher, the editors and the reviewers. Any product that may be evaluated in this article, or claim that may be made by its manufacturer, is not guaranteed or endorsed by the publisher.
